# Case Report: Neonatal genital prolapse in a pair of premature Twins

**DOI:** 10.3389/fped.2023.1294348

**Published:** 2024-01-09

**Authors:** Jian Gu, Xiaochun Chen, Shikun Li, Wenliang Chen, Xinqi Zhong

**Affiliations:** ^1^ Department of Neonatology, Department of Obstetrics and Gynecology, The Third Affiliated Hospital of Guangzhou Medical University, Guangzhou, China; ^2^ Guangdong Provincial Key Laboratory of Major Obstetric Diseases, Guangdong Pronvincial Clinical Research Center for Obstetrics and Gynecology, Guangzhou, China; ^3^ Department of Pediatrics, The Third Affiliated Hospital of Guangzhou Medical University, Guangzhou, China; ^4^ Scientific Research Center, The Second Affiliated Hospital of Guangdong Medical University, Zhanjiang, China

**Keywords:** preterm infant, twin, neonatal genital prolapse, selective intrauterine growth restriction, growth retardation

## Abstract

Neonatal genital prolapse is a rare situation. This report presents a unique case involving a pair of premature female twins who both developed vaginal wall prolapse without any neurological deficits. Multiple factors such as selective intrauterine growth restriction, feeding intolerance, extrauterine growth retardation, and elevated intra-abdominal pressure after birth may have contributed to the development of this phenomenon. Notably, the severity of prolapse was more pronounced in the twin with lower birth weight and smaller for gestational age. After a five-month follow-up period, the twins’ prolapsed vaginal wall fully retracted due to a combination of conservative treatment and enhanced nutritional support.

## Introduction

Neonatal genital prolapse (NGP) is a rare disorder, which is often associated with spinal bifida, but it can also manifest in the absence of any underlying defects (1, 2). The description of NGP firstly concentrated in term infants and then sporadic cases of NGP in premature infants were reported, but all of them were singleton pregnancy (3, 4). No reports of twins about NGP have been retrieved. This is the first case reported the premature twins who experienced genital prolapse despite the absence of neurological deficits.

## Case report

The monochorionic twins were delivered via cesarean section at 34 weeks of gestation due to the selective intrauterine growth restriction. This was the mother's first naturally conceived pregnancy, during which she refrained from smoking or exposure to harmful substances. Both twins are female, displayed a weight disparity of 36% at birth, with the second-born twin being smaller (1,130 g, <3rd percentile). Upon admission to the neonatal intensive care unit, no conspicuous physical anomalies were noted although the labia majora did not fully cover the labia minora, consistent with other preterm infants. The twins were treated with the continuous positive airway pressure ventilation (for less than 7 days) without needing steroids during their hospital stay.

**Figure 1:**
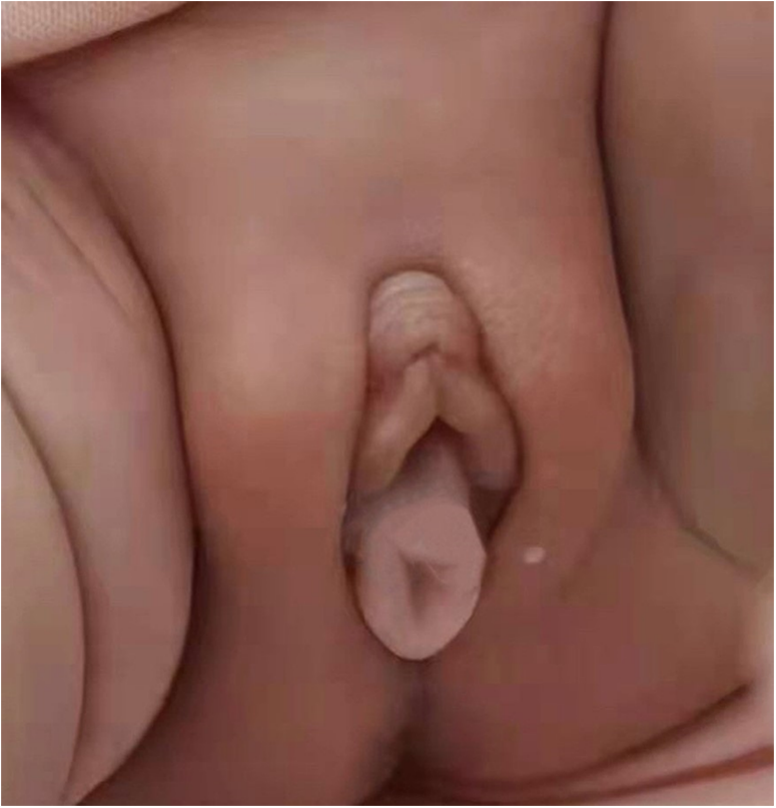
The perineum of first-born twin.

**Figure 2:**
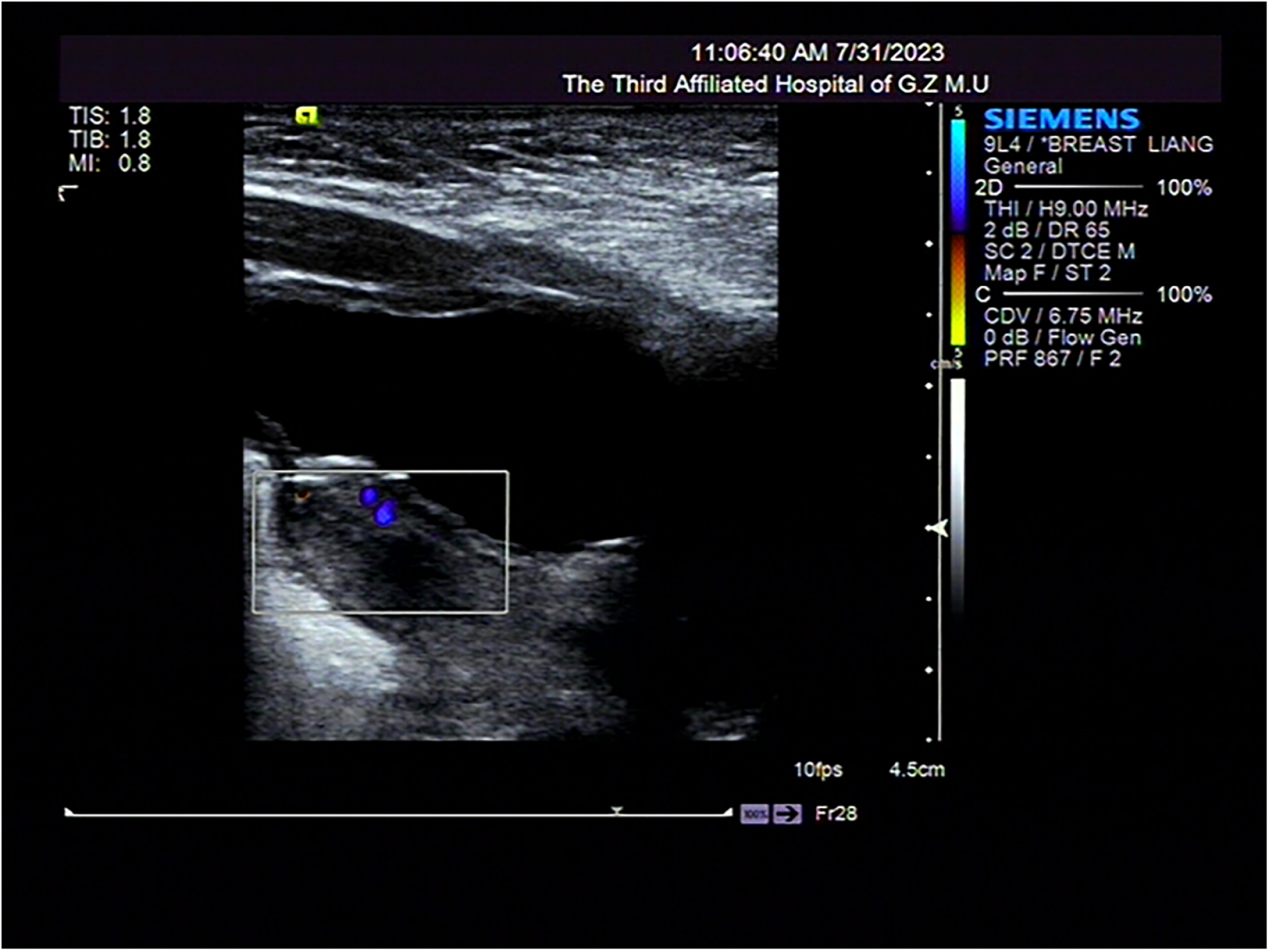
Pelvic and abdominal ultrassonography imaging of the first-born twin.

**Figure 3:**
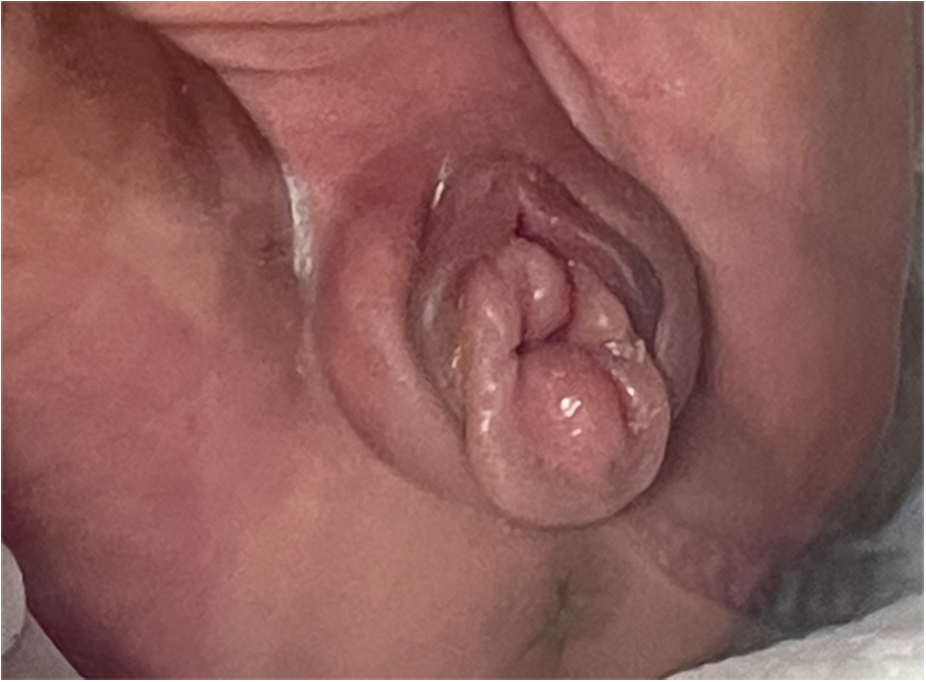
The perineum of second-born twin.

**Figure 4:**
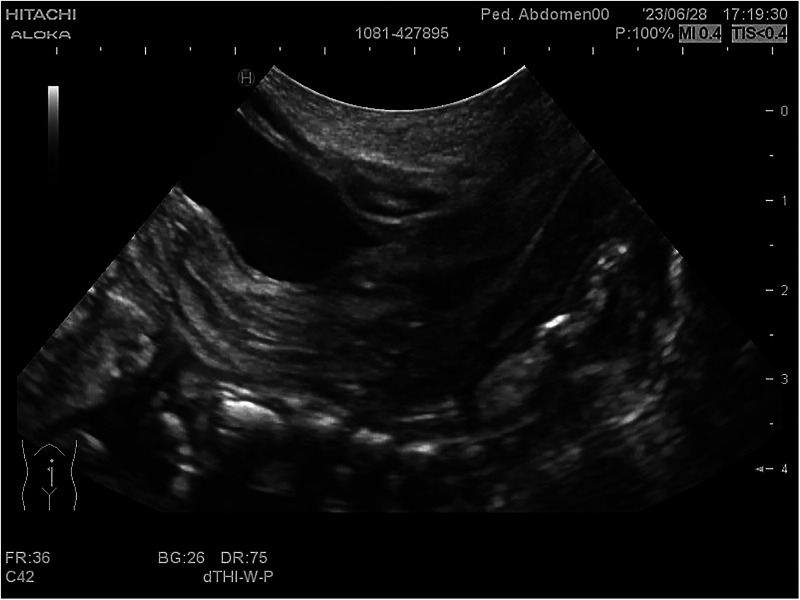
Pelvic and abdominal ultrassonography imaging of the second-born twin.

Necrotizing enterocolitis was suspected in both twins because of their inability to tolerate feed and the repeated occurrence of bloody stools. As a result, it was decided to withhold oral feeding three days before gradually resuming enteral feeds. Persistent abdominal distension persisted during this period. Moreover, the weight gain of twins is slow, less than 3rd percentile when corrected for gestational age at full term. On the 40th day after the birth of the first-born twin and the 43rd day after the second-born twin's birth, a light red, soft tissue protrusion became evident in the perineal area. The distal end of the prolapsed mass was clearly visible at the the perineal orifice (Figures 1, 3). The urethral meatus was in a normal position and the twins urinated normally. Pelvic and abdominal ultrassonography revealed no abnormalities in the position of the uterine body or cervix, and no prominent mass echoes were observed in the bilateral adnexal region. The anomalous lesions extending from the vaginal wall were diagnosed as vaginal wall prolapse (Figures 2, 4). On routine cranial ultrasound a subependymal hemorrhage was detected, a spinal x-ray, as well as an magnetic resonance imaging(MRI) under oral chloral of head, lumbar spine and thoracic spine, revealed no evidence of congenital neuromuscular conditions. Besides, neither Neonatal Behavioral Neurological Assessment score nor Test of Infant Motor Performance score showed significant abnormalities. Under the guidance of gynecologists, attempts were made for the prolapsed vaginal wall by manual reduction. However, the vaginal wall rapidly returned to its prolapsed state upon the release of pressure. The vaginal wall prolapsed became pronounced when the twins cried. The vaginal wall could not autonomously return to its original position even though the twins calmed down.

In the absence of urinary obstruction, and given the lack of increasing abdominal distension or any apparent redness or swelling at the vaginal opening of the vulva, a conservative approach was chosen for treatment. This involved daily manual reduction and intensified nursing care, which included emphasizing local cleanliness and avoiding factors that might increase abdominal pressure. Additionally, considering the twins’ slow weight gain and concurrent extrauterine growth retardation, efforts were made to enhance their daily nutrition supply with the aim of promoting weight gain.

After their hospital discharge, regular outpatient follow-up was performed for the twins. In the case of the first-born twin, after a 5-month interval, her weight retrieved to 7.2 kg (>50th percentile), accompanied by a body length of 64 cm (>50th percentile) and a head circumference of 42 cm (>50th percentile). The prolapsed vaginal wall was completely retracted. Similarly, for the second-born twin, whose prolapse was more severe, at the 5th month follow-up after discharge, her weight was 5.3 kg (>3rd percentiles), accompanied by a length of 58.5 cm (>3rd percentiles) and a head circumference of 39.5 cm (>10th percentiles). At achieving weight pursuit, the prolapsed vaginal wall was also fully retracted.

## Discussion

Genital prolapse is more common in elderly women or multipara, often due to pelvic floor muscle relaxation (5). However, this disorder is rare in neonates, and there is no report of genital prolapse in twins currently. NGP usually performs as a light red, soft tissue protrusion from the perineum, ranging from mild vaginal protrusion to a complete prolapse involving the cervix and uterus. In cases of vaginal wall prolapse, the prolapsed mass is usually circumferential. Furthermore, the external opening of the cervix can often be seen at the upper end of the prolapse mass with unaffected urethral opening (6, 7).

Approximately 80% cases of NGP are related to spinal cord malformations and lack of pelvic floor muscle strength. Subsequently, the etiological factors for genital prolapse have been categorized into two subgroups. The first subgroup are cases with abnormal neurological manifestations, such as spina bifida and meningomyelocele. Another etiological subgroup is cases with normal neuroanatomy, which have diverse causes like labor injuries during delivery or increased intra-abdominal pressure (4, 8–10). In cases of mild prolapse, some reports have shown that they were not accompanied with congenital neurological abnormalities (11, 12). Other reasons such as intrauterine malnutrition, long-term feeding intolerance, prolonged hormone therapy, fluid restriction, and elevated intra-abdominal pressure also have been indicated relevant to the happening of prolapse (4, 13).

For the treatment of neonatal genital prolapse, the most common used method is manual reduction, showing favorable outcomes for cases of mild prolapse and with no relapse during follow-up (11). However, there is a tendency for recurrence constantly after manual reduction in some cases, especially in infants with moderate prolapse. In such instances, utilizing Foley silicone catheters or hypertonic saline packs to provide rudimentary uterine support may help in preventing prolapse (7, 14, 15). Nonetheless, if the capacity of conservative therapy to sustain the original genital position is limited and it potentially impeded lower limb perfusion circulation (16), we should take other alternative interventions such as uterine suspension surgery, partial labial fusion surgery, and purse string suture to rectify the prolapse (16–18). However, some experts advocate that the more aggressive surgical approaches could be executed only when the conservative treatments fail or the persistent recurrent prolapse accompanied by changes of hypertrophy and ulceration in vaginal mucosal (14).

The twins described in this case report didn't have congenital nervous system abnormalities. Although both the them suffered the selective intrauterine growth restriction during their mother's pregnancy, it is noteworthy that the second-born twin, a smaller for gestational age preterm infant, experienced a more severer form of prolapse. Morever, the twins encountered similar issues such as abdominal distension, feeding intolerance and extrauterine growth retardation after birth. As we progressively enhanced their nutritional support and facilitated weight gain, favorable outcomes were observed in the resolution of vaginal prolapse.

Remarkably, this was the first time to report the case of genital prolapse in premature twins. We speculate that multifactors including intrauterine malnutrition, being smaller for gestational age, experiencing extrauterine growth retardation and confronting elevated abdominal pressure may collectively contribute to the onset of NGP. Importantly, with removal of these risk factors, the scenario of genital prolapse can spontaneously ameliorate.

## Data Availability

The original contributions presented in the study are included in the article/Supplementary Material, further inquiries can be directed to the corresponding authors.
